# The effect of family continuity management on the anxiety level of parents of children with febrile seizures and recurrence rate

**DOI:** 10.3389/fmed.2026.1773168

**Published:** 2026-04-10

**Authors:** Xiali Xu, Yanyan Chen, Lingyu Zhong, Li Hong, Xia Jin, Han Miao, Lingling Feng, Peng Bai, Dongmei Xu, Rongrong Chen

**Affiliations:** Department of Pediatrics, Affiliated Maternity and Child Health Care Hospital of Nantong University, Nantong Children's Hospital, Nantong, Jiangsu, China

**Keywords:** anxiety level, family continuity management, febrile seizures, parental anxiety, recurrence rate

## Abstract

**Objective:**

Analyze the impact of family continuity management on the anxiety level of parents of children with febrile seizures and recurrence rate.

**Methods:**

One hundred and fourteen children with febrile seizures were selected and divided randomly into a control group (*n* = 57, with routine discharge guidance intervention) and a study group (*n* = 57, with family continuity management intervention) by random number table method. The clinical indicators, complication rates, recurrence rates and psychological states of parents [assessed by Self-Rating Anxiety Scale (SAS) and Self-Rating Depression Scale (SDS)] of 2 groups were compared.

**Results:**

The emergency time, fever reduction time, hospitalization time, seizure control and disappearance time of study group were all shorter than control group (*P* < 0.05). After intervention, SDS and SAS scores of parents of both groups of children decreased, the parents of children in study group were lower than control group (*P* < 0.05). During the 6 month follow-up period, the incidence of complications and recurrence rates in study group were lower than control group (*P* < 0.05).

**Conclusion:**

Family continuity management can shorten the time for symptoms to disappear in children with febrile seizures, reduce parental anxiety levels, and also has good value in preventing complications and recurrence.

## Introduction

Febrile seizures are common in children aged 6 months to 5 years. Their typical manifestations are sudden, brief, tonic, or clonic convulsions of the whole body or local muscle groups, often accompanied by loss of consciousness (1). This disease has a sudden onset and progresses rapidly, so it is very necessary to take proper first aid measures.

However, parents of the children often have insufficient understanding of the disease and feel anxious and fearful about their children's safety. These negative psychological states will directly affect the parents' ability to care for their children. If parents take improper measures to intervene or delay seeking medical treatment when the child falls ill, it will directly affect the first aid effect and even pose a threat to the child's life safety. One study (2) have shown that the first experience of witnessing a child's febrile convulsion has a huge psychological impact on parents, often plunging them into extreme fear and inducing anxiety. Another research (3) indicates that although most children have good treatment outcomes, the disease has a tendency to recur, with a recurrence rate as high as 30% to 50%. The repeated occurrence of convulsions not only increases the anxiety level of parents but may also lead to significant depressive and post-traumatic stress disorder-like symptoms, and even induce a decline in the family's overall quality of life. Therefore, family health education and care for children after discharge are crucial for reducing the recurrence rate, improving the prognosis of the children, and alleviating the psychological burden on parents.

Continuity of care intervention is an extension of care from the hospital to the home, and its effectiveness in the rehabilitation of chronic diseases has been confirmed (4). Due to the characteristics of febrile seizures, such as suddenness of occurrence, intermittence, and relatively concentrated care knowledge, a targeted continuous care plan needs to be formulated grounded on the specific needs of the children's families and the characteristics of febrile seizures. At present, there are already relatively abundant studies on the application of family continuous care in children with febrile convulsions. The intervention measures of the existing plans mostly focus on health education and regular follow-up after discharge, with the main contents being guidance on disease awareness and first aid skills. In terms of research design, most studies adopt quasi-experimental designs, lacking strict randomized controlled trials, and the sample sizes are generally small with short follow-up periods. In terms of measurement results, existing studies mainly focus on objective indicators such as the fever rate of children patients, the recurrence rate of convulsions, parents' mastery of first aid knowledge, and nursing satisfaction. However, the measurement of parents' psychological aspects is relatively insufficient, and few studies have systematically evaluated parents' anxiety as a core outcome indicator. As the main executors of continuing care, parents' anxiety not only affects their own health but may also directly impact the quality of the implementation of care measures.

Therefore, this research on the basis of conventional continue nursing increase psychological support intervention for parents anxiety depression, through quantitative parents before and after the intervention of children with anxiety, depression scores and prognosis index, system of family continue nursing to alleviate parents anxiety and improve the prognosis of children with dual effect, to make up for the inadequacy of existing research concern for parents psychological level, In order to provide a reference basis for constructing a continuous nursing program with a dual core of “child patients–parents.”

## Materials and methods

### General information

A total of 114 children with febrile seizures in our hospital from December 2021 to December 2024 were selected. Eligible participants were randomly assigned to the study or control group, with 57 cases in each group. The randomization sequence was computer-generated by an independent statistician who was not involved in participant recruitment, enrollment, or intervention delivery. Computer-generated block randomization with variable block sizes of 4 and 6 were used to ensure equal distribution between the two groups. The allocation sequence was concealed using sequentially numbered, opaque, sealed envelopes. Envelopes were opened only after the participant's baseline data were collected. Outcome assessors were blinded to group assignment. Data on clinical indicators, complications, and recurrence were extracted from electronic medical records by research assistants who were not involved in the intervention delivery. Basic conditions of 2 groups were comparable (*P* > 0.05), as given in Table 1.

**Table 1:** Comparison of basic data of 2 groups of children [*n* (%), $\bar{x}\pm s$].

Groups		Study group (*n* = 57)	Control group (*n* = 57)	*t*/χ^2^ value	*P* value	*t* value	*df* value	z value	95% confidence interval
Age (months)		23.75 ± 8.74	24.08 ± 9.03	0.822	0.412	0.1983	112		−3.628~2.968
Gender	Male	32 (56.14)	30 (52.63)	0.181	0.669		1	0.3761	
	Female	25 (43.86)	27 (47.37)						
First body temperature (°C)		39.22 ± 0.61	39.06 ± 0.72	1.674	0.098	1.280	112		−0.08765~0.4077
History of previous febrile seizures	Yes	18 (0.316)	20 (0.351)	0.1579	0.6911		1	0.3974	
	No	39 (0.684)	37 (0.649)						
Caregiver's age	< 35	35 (0.614)	36 (0.632)	0.1579	0.6911		1	0.3974	
	≥35	22 (0.386)	21 (0.368)						
Caregiver education level	High school and below	19 (0.333)	18 (0.316)	0.04001	0.8425		1	0.2000	
	Associate degree or above	38 (0.667)	39 (0.684)						
Family monthly income	≤ 5000	7 (0.123)	8 (0.140)	0.1614	0.9225		2		
	5000~15000	29 (0.509)	27 (0.474)						
	≥15000	21 (0.368)	22 (0.386)						

Inclusion criteria: ① In accordance with the Consensus Statement on the Diagnosis and Management of Febrile Seizures (5); ② Age 6 months to 5 years; ③ Generalized tonic-clonic seizures induced by rectal temperature ≥ 38 °C; ④ The main caregiver was the parents, they all signed the informed consent form.

Exclusion criteria: ① Complex febrile seizures or underlying neurological diseases; ② Complicated with severe heart, lung, kidney or liver diseases; ③ Without a fixed guardian accompanying; ④ Guardians had mental disorders or communication barriers, and were unable to cooperate with the follow-up; ⑤ Central nervous system infections, metabolic abnormalities, and epilepsy history; This research got approval of our hospital's medical ethics committee. Total 156 children with febrile seizures were initially screened for eligibility in 3 years. Of these, 42 patients were excluded for the following reasons: complex febrile seizures or underlying neurological diseases (*N* = 18), central nervous system infections, metabolic abnormalities, or epilepsy history (*N* = 10), complicated with severe heart, lung, kidney, or liver diseases (*N* = 6), Without a fixed guardian accompanying (*N* = 5), and guardians had mental disorders or communication barriers, unable to cooperate with follow-up (*N* = 3).

### Intervention methods

Control Group: Routine discharge guidance intervention. At the time of discharge, oral education was provided. The responsible nurse explained the knowledge of febrile seizures to the parents of the children, including the pathogenesis and family handling principles; a uniformly printed “Family Care Manual for Febrile Seizures” was distributed; the follow-up time at the outpatient clinic was informed. After discharge, a telephone follow-up was conducted once every 4 weeks, but no continuous care was provided.

Study Group: Family Continuity Management Intervention, lasting for 6 months. (1) Structured health education was initiated before discharge, lasting for 30 min. The parents were explained the essence of the disease, and the mechanism of convulsions and the favorable prognosis were analyzed through animated videos; high-risk factors for recurrence and prevention of recurrence were explained; the use of electronic thermometers and the proper usage of antipyretic drugs were demonstrated; the Checklist for Dealing with febrile seizures was distributed, and the emergency procedures were provided. Under the guidance of the nurses, parents simulated the handling of seizure episodes and practiced the skills through practical exercises. (2) Continuation intervention after discharge. A dedicated communication platform was established, and a family continuity management team was formed, consisting of 1 specialist doctor and 2 nurses. A WeChat management group was set up, and the parents of the children of study group were invited. Online Q&A was conducted from 9:00 to 17:00 on weekdays, and 1 educational short video on common misconceptions about physical cooling and fever diet guidelines was pushed out every week. Telephone follow-up plan: Within 3 days after discharge, a telephone follow-up was conducted to assess the parents' mastery of the operation, their anxiety level, and to answer their questions; on the day of the fever event, remote guidance was provided on the fever-reducing strategy and risk assessment of recurrence; and a telephone follow-up was conducted every 4 weeks to reinforce the parents' disease knowledge, soothe their psychology, and remind them of the need for a follow-up visit. A family emergency kit was also provided, including 1 electronic ear thermometer, a first aid card, 10 pieces of fever-reducing patches, and 2 dose syringes. (3) Periodic reinforcement. During the 1-month follow-up visit, the nurse conducted on-site assessments of parents' skills in temperature monitoring and medication preparation; the Self-Rating Anxiety Scale (SAS) was distributed to value the psychological state (6). At the 3-month mark, a group discussion session was organized, where parents shared their coping experiences with each other; a psychological therapist taught the mindfulness breathing relaxation technique to alleviate their psychological burden.

**Figure 1:**
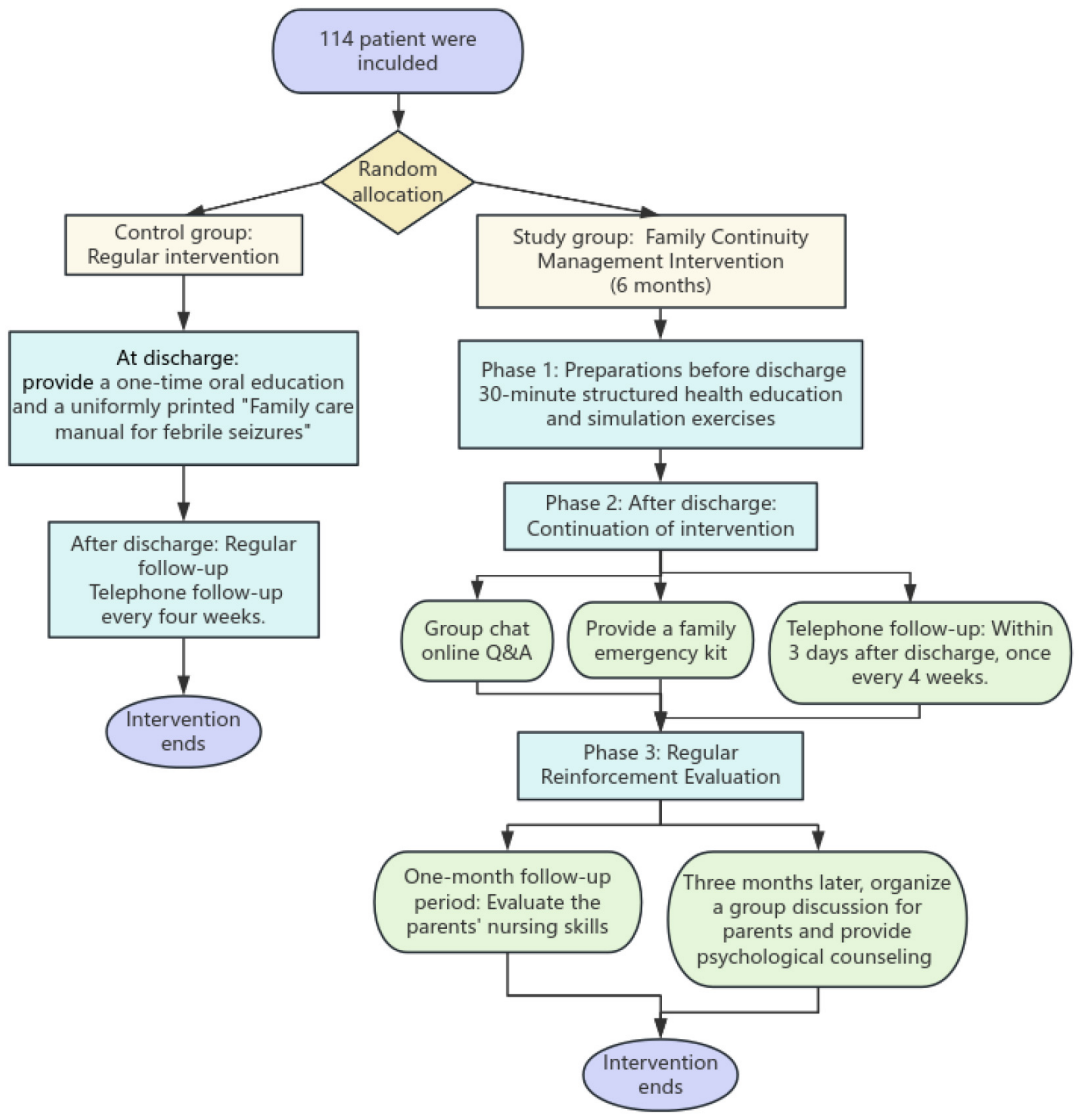
Schematic diagram of the two groups of intervention measures.

The intervention measures for the 2 groups of patients and their parents are exhibited in Figure 1. All intervention sessions were delivered by the same team of trained nurses using standardized educational materials and checklists.

### Observation indicators

(1) Clinical Indicators: Record the emergency treatment time, fever reduction time, seizure control time, seizure disappearance time, and hospitalization time for both groups of children. (2) Parents' Psychological State: The anxiety and fear of parents are significant obstacles and important outcome indicators for the family management of children with febrile seizures, and this has been confirmed by numerous studies (7). In this research, the SAS and the Self-Rating Depression Scale (SDS) (8) were used to quantitatively assess the effect of intervention measures on alleviating the psychological stress of parents (9). Before and after the intervention, use SDS and SAS to evaluate parents' psychological state. Both scales have 20 items, scored from 1 to 4. The total score of each item is added to obtain the raw score. Both scales have a maximum score of 80, and multiplied by 1.25 and then rounded to get the standard score. The lower the score, the less severe the parents' anxiety and depression. (3) Complications: In the 6-month follow-up period, observe the occurrence of complications in both groups of children, including tongue laceration, dehydration, and aspiration pneumonia, etc. (4) Recurrence Rate: During the 6-month follow-up period, observe the recurrence of the children' conditions. Criteria: Fever ≥ 38°C, with seizure episodes, and excluding other causes, is considered as recurrence of the condition.

TIDieR and CONSORT 2010 Checklist Mapping were provided in Supplementary file.

### Statistical methods

Data were processed utilizing SPSS 26.0. Quantitative data were presented as ($\bar{x}\pm s$) and were analyzed by *t*-test; qualitative data were calculated as proportions and analyzed employing χ^2^ test. Differences were considered explorative significant when *P* < 0.05.

## Results

### Comparison of clinical indicators between the two groups

The Emergency response time, fever reduction time, seizure control and disappearance time, hospitalization time of patient children in study group were all shorter than control group (*P* < 0.05, Table 2).

**Table 2:** Comparison of clinical indicators between 2 groups [$\bar{x}\pm s$].

Groups	Emergency response time (min)	Fever reduction time (h)	Duration of seizure control (min)	Duration of seizure disappearance (d)	Hospital stay (d)
Study group (*n* = 57)	24.53 ± 4.82	28.04 ± 1.42	2.53 ± 0.47	3.98 ± 0.27	5.48 ± 1.26
Control group (*n* = 57)	40.01 ± 5.77	44.65 ± 4.18	5.57 ± 0.62	6.17 ± 1.04	9.43 ± 1.48
*t* value	15.545	28.406	29.500	15.388	15.343
*P* value	< 0.001	< 0.001	< 0.001	< 0.001	< 0.001
*df* value	112	112	112	112	112
95% confidence interval	−17.45~-13.51	−17.77~-15.45	−3.244~-2.836	−2.472~-1.908	−4.460~-3.440

### Comparison of psychological states of parents of the two groups

After intervention, SAS and SDS scores of parents of both groups of children decreased, in terms of parents' scores, the study group were lower than control group (*P* < 0.05, Table 3).

**Table 3:** Comparison of psychological states of parents of 2 groups [score, $\bar{x}\pm s$].

Groups	SAS score		SDS score	
	Before intervention	After intervention	Before intervention	After intervention
Study group (*n* = 57)	54.76 ± 4.57	42.77 ± 3.74^*^	51.82 ± 4.71	40.87 ± 4.13^*^
Control group (*n* = 57)	55.18 ± 4.83	47.95 ± 4.16^*^	52.24 ± 4.89	45.22 ± 4.36^*^
*t* value	0.477	6.991	0.467	5.469
*P* value	0.634	< 0.001	0.641	< 0.001
*df* value	112	112	112	112
95% confidence interval	−2.165~1.325	−6.648~-3.712	−2.202~1.362	−5.926~-2.774

Compared with the baseline values of the same group,

^*^P < 0.05.

### Comparison of complications between the two groups

The complication rate of these patient children of study group during the 6-month follow-up period was lower than control group (*P* < 0.05, Table 4).

**Table 4:** Comparison of complications between 2 Groups [*n* (%)].

Groups	Tongue bite	Dehydration	Aspiration pneumonia	Total cases
Study group (*n* = 57)	0 (0.00)	2 (3.51)	0 (0.00)	2 (3.51)
Control group (*n* = 57)	4 (7.02)	4 (7.02)	3 (5.26)	11 (19.30)
χ^2^ value				7.033
*P* value				0.008

### Comparison of recurrence rates between the two groups

During the 6-month follow-up period, 3 cases (5.26%) of recurrence occurred in study group, while 11 cases (19.30%) occurred in control group. A significant difference in the recurrence rates was seen between 2 groups (χ^2^ = 5.221, *P* = 0.022, Table 5).

**Table 5:** Comparison of recurrence rates between 2 groups [*n* (%)].

Groups	Recurrence rates
Study group (*n* = 57)	3 (5.26)
Control group (*n* = 57)	11 (19.30)
χ^2^ value	5.221
*P* value	0.022

## Discussion

Studies (10) have shown that factors such as age, gender, and genetics are closely related to the occurrence of febrile seizures, and in severe cases, they can develop into epilepsy, affecting the prognosis of the children. Therefore, implementing health education for parents to enhance their knowledge and coping strategies is beneficial for controlling the occurrence and development of the disease. This research, through the implementation of family continuity management intervention, found that clinical indicators of children of study group were better than control group, suggesting that family continuity management is conducive to the disappearance of symptoms, thereby facilitating the recovery of the children. A study has indicated (11) that continuous care can reduce the risk of nursing problems, optimize parents' nursing knowledge and health behaviors, and enhance the immunity of children and reduce the recurrence rate. Relevant studies have shown (12, 13) that continuous care can provide effective health education for parents of children with febrile seizures and significantly shorten the time for emergency treatment, hospitalization, and symptom disappearance. Continuous management could help parents effectively acquire disease-related knowledge, improve their health behaviors, enable them to effectively observe the child's condition, provide appropriate family care for the child, promote the improvement of the child's condition, thereby achieving the desired nursing effect.

A study has indicated (14) that the correct first-aid measures taken by parents during a febrile seizure are crucial, and this is closely related to the parents' knowledge of the disease and their psychological state. In this study, after the intervention, the SAS and SDS scores of the parents in the study group were lower than control group, suggesting that family continuous management can improve the parents' psychological state, which is similar to the research results of Almousa et al. (15). The root cause of parents' anxiety lies in misunderstandings of the disease essence and knowledge gaps. Studies (16) have shown that structured and standardized health education can help parents reduce misunderstandings about diseases, thereby lowering their anxiety levels. This indicates that through systematic health education and multi-channel reinforcement, it is possible to effectively correct parents' incorrect perceptions and alleviate their anxiety. Meanwhile, simulation training has also been proven to enhance parents' confidence in practical operations and further reduce anxiety (17). This indicates that practical exercises can help improve the standardization level of guardians' caregiving behaviors and enhance their psychological state by boosting self-efficacy. Research (18) has proved that providing parents with a 24 h consultation hotline can reduce their helplessness when facing unexpected situations at night and alleviate the psychological impact of stressful events. This shows that a dynamic support network can provide comprehensive support for parents, interrupt the fear-error handling chain of parents, and the online guidance of medical staff can correct family care misunderstandings, avoid improper operations that trigger secondary anxiety, and thereby improve their psychological state.

Furthermore, studies have confirmed (19) that for each recurrence of the child's condition, the risk of parental anxiety increases by 2.1 times. In this study, the incidence of complications and recurrence rate of study group during the follow-up period were both lower than control group, suggesting that family continuous management can reduce the occurrence and recurrence risks of complications in children. A study has shown (20) that the effect of reducing complications on alleviating anxiety is even higher than direct psychological intervention. Parents who have received standardized first aid training can protect the children, reduce secondary injuries caused by complications, and directly alleviate their feelings of guilt, self-blame, and anxiety. This is mainly because parents who have received standardized first aid training can reduce incidents related to convulsions, prevent secondary harm from complications, directly weaken the parents' sense of guilt and self-blame, and anxiety. Additionally, during the continuous management period, the research team effectively enhanced parents' understanding of the disease and their ability to provide family care by offering them scientific health knowledge education and conducting online Q&A sessions through WeChat groups. This provided an important guarantee for reducing the recurrence risk in the children.

The study involved only two groups (study vs. control), and multiple independent statistical tests (*t*-tests and chi-square tests) were performed on several distinct, pre-specified outcome measures (e.g., clinical time parameters, psychological scores, complication rate, recurrence rate). This may constitute a multiple testing scenario. This study was designed as an exploratory investigation aiming to provide a comprehensive, preliminary assessment of the potential effects of the Family Continuity Management intervention across different, clinically relevant dimensions. Each outcome was selected *a priori* based on its clinical and theoretical importance, and they represent distinct domains of effect (efficacy, safety, psychological impact). In such exploratory contexts within nursing and behavioral intervention research, it is a common practice to report unadjusted *p*-values for pre-specified outcomes to avoid over-correction and potential Type II errors, thereby generating hypotheses for future confirmatory trials.

It should be noted that the main innovative attempts of this study lie in the following: Firstly, this study breaks away from the previous practice of starting family continuation management intervention only after the child is discharged from the hospital. Instead, health education and first aid training are conducted before discharge, aiming to enhance parents' awareness of febrile seizures and their ability to handle emergencies. Secondly, this study differs from previous studies that only focused on the prognosis of children with high fever seizures. It incorporates the use of SAS and SDS scales to assess the psychological status of caregivers as a core outcome indicator into the evaluation system for the effectiveness of family continuation care. This expansion provides a new analytical framework for subsequent research to shift from a “child-centered” approach to a “family-centered” one. Thirdly, within the regular continuation care plan, this study specifically incorporates modules for assessing parents' anxiety and providing psychological support. It innovatively conducts group discussions during the follow-up period to offer psychological counseling courses to parents, effectively reducing their psychological stress. A “child-parent” dual-core intervention model has been constructed. The research results suggest that this model has certain application value in improving the prognosis of children and alleviating parents' anxiety, providing a reference basis for clinical nurses to optimize continuation care plans.

This study has several limitations that should be acknowledged. First, as a single-center trial with a relatively small sample size and no *a priori* power calculation, the generalizability of findings is limited and the study may have been underpowered to detect smaller effects. Second, the 6-month follow-up period, though adequate for assessing short-term recurrence, is insufficient to evaluate long-term neurological outcomes or sustained psychological impact. Third, potential confounding factors including socioeconomic status, parental education level, and detailed family environment were not systematically controlled despite baseline comparability. Finally, the intervention's reliance on a WeChat-based platform and its resource-intensive components (specialist team, simulation training, and emergency kits) may limit applicability in settings with low digital access or limited healthcare resources. Future multicenter studies with larger samples, longer follow-up, blinded assessment where feasible, and formal implementation research are needed to validate and refine this approach.

In conclusion, family continuity management can shorten the time for children's symptoms to disappear, reduce parents' anxiety levels, and also has good effects in preventing complications and reducing recurrence. However, due to the scarcity of clinical-related studies, the clinical application value of family continuity management for children with febrile seizures still needs to be further explored and analyzed.

## Data Availability

The original contributions presented in the study are included in the article/supplementary material, further inquiries can be directed to the corresponding author.
